# The assessment of the sexual functioning in women with idiopathic scoliosis- preliminary study

**DOI:** 10.1186/1748-7161-9-S1-O82

**Published:** 2014-12-04

**Authors:** Jacek Durmala, Irmina Blicharska, Agnieszka Drosdzol-Cop, Violetta Skrzypulec-Plinta

**Affiliations:** 1 Department of Rehabilitation, Medical University of Silesia, Katowice, Poland; 2 Women’s Health Institute, School of Health Sciences Medical University of Silesia, Katowice, Poland

## Background

Sexuality is conditioned by the integrated action of biological, psychological and socio-cultural factors. Inhibition or distortion of this function, as well as disharmony with other spheres of human personality is causing the sexual dysfunction. Idiopathic scoliosis (IS) manifested many deformities in the trunk often occurs in periods of change in the development of the child psyche and personality. Disorders affecting the physical realm, especially the appearance of the body impact on perception of their differences, lack of acceptance and reduce self-esteem. All this may lead to the functioning disorders of the many areas of life, including sexuality.

The aim of the study was to determine the occurrence frequency of sexual dysfunctions in women with IS and to find a correlation with scoliotic deformation.

## Design

Prospective, randomized and double blind studies.

## Methods

In a study participated 36 women aged 18-24 years (mean 20.7±1.9) with IS. Sexual functioning of respondents was evaluated using a standardized questionnaire Female Sexual Functional Index (FSFI). The survey questions related to sexual activity in the previous four weeks, including her 6 domains: desire, arousal, lubrication, orgasm, satisfaction and pain. The value of the total score below 26 points indicates the presence of dysfunction in the sexual sphere. On the basis of the current X-ray, the values of Cobb and AVR were determined.

## Results

Average results of FSFI score including the individual domains were as follows: desire-3.72, arousal-3.49, lubrication-4.03, orgasm-3.43, sexual satisfaction-4.01, pain associated sexual intercourse-3.43. Among the respondents, 34.28% of women received total score indicating the presence of sexual dysfunction clinical significantly symptoms. The average value of points obtained in the group was 22.1±2.46. There was no correlation between FSFI test result and the Cobb and AVR primary curvature values (p> 0.05).

## Conclusions

1. Based on this evaluation it can be concluded that women with IS have problems with sexual functioning, for the most experience orgasm and pain during sexual intercourse.

2. It is necessary further research and to extend their scope to search for factors influencing on the disorders occurrence and to determine their relationship with the scoliosis occurrence.

